# Exploring Cell Tropism as a Possible Contributor to Influenza Infection Severity

**DOI:** 10.1371/journal.pone.0013811

**Published:** 2010-11-23

**Authors:** Hana M. Dobrovolny, Marc J. Baron, Ronald Gieschke, Brian E. Davies, Nelson L. Jumbe, Catherine A. A. Beauchemin

**Affiliations:** 1 Department of Physics, Ryerson University, Toronto, Ontario, Canada; 2 F. Hoffmann-La Roche Ltd, Basel, Switzerland; 3 F. Hoffmann-La Roche, Inc., Nutley, New Jersey, United States of America; University of Georgia, United States of America

## Abstract

Several mechanisms have been proposed to account for the marked increase in severity of human infections with avian compared to human influenza strains, including increased cytokine expression, poor immune response, and differences in target cell receptor affinity. Here, the potential effect of target cell tropism on disease severity is studied using a mathematical model for in-host influenza viral infection in a cell population consisting of two different cell types. The two cell types differ only in their susceptibility to infection and rate of virus production. We show the existence of a parameter regime which is characterized by high viral loads sustained long after the onset of infection. This finding suggests that differences in cell tropism between influenza strains could be sufficient to cause significant differences in viral titer profiles, similar to those observed in infections with certain strains of influenza A virus. The two target cell mathematical model offers good agreement with experimental data from severe influenza infections, as does the usual, single target cell model albeit with biologically unrealistic parameters. Both models predict that while neuraminidase inhibitors and adamantanes are only effective when administered early to treat an uncomplicated seasonal infection, they can be effective against more severe influenza infections even when administered late.

## Introduction

The potential spread of a severe pandemic influenza is a worldwide cause for concern. In recent years, attention has focused on the avian-derived influenza A (H5N1) virus strain, which has the potential to evolve into a pandemic influenza strain [1]. The swine-origin influenza A (H1N1) strain which is responsible for the recent influenza pandemic has been a cause for concern given the strain's ability to cause severe illness and the added stress it puts on the health care system [2]–[6]. The reasons for the increased severity observed with some influenza strains are poorly understood and possible explanations include an excessive cytokine response [7]–[11], a poor immune response due to the strain's novelty [12], [13], and differences in target cell receptor affinity (cell tropism) between human-adapted, seasonal strains and animal-origin pandemic strains [14]–[17]. Recent work has focused on the binding affinity of different strains of influenza virus for specific cell receptors within the respiratory tract [18]–[21] and it is believed that this difference in affinity between human and avian strains may in part be responsible for the difference in severity between the two strains, though the reasons for this are currently not well understood.

Two specific cell types are believed to play important roles in influenza virus infection: ciliated epithelial cells, and nonciliated, mucus-producing cells. In epithelial cell cultures, nonciliated, mucus producing cells predominantly express sialic acid 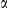-2,6 galactose terminated saccharides (SA
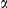
2,6 Gal) on their surface, while ciliated cells express sialic acid 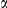-2,3 galactose terminated saccharides (SA
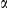
2,3 Gal) receptors, as well as SA
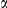
2,6 Gal receptors, on their surface [20], [22], [23]. In vitro experiments have shown that human-adapted influenza A viruses (H1N1, H3N2) seem to preferentially bind to SA
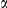
2,6 Gal receptors, while avian-adapted influenza A (H5N1) viruses appear to preferentially bind to SA
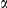
2,3 Gal cell receptors [16]. Due to concerns over the effect of cell tropism on infection dynamics, most influenza infection assays are now conducted in Madin-Darby canine kidney (MDCK) cells which have been transfected to express more SA
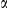
2,6 Gal receptors (called SIATI cells), rather than in regular MDCKs which predominantly express SA
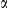
2,3 Gal receptors [24]. A similar trend has developed for in vivo influenza infection assays which are now preferably performed in ferrets rather than mice because the former has lung cells which predominantly express (human lung-like) SA
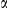
2,6 Gal receptors, while the latter mostly has lung cells expressing SA
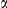
2,3 Gal receptors [25]–[27]. The adoption of ferret models for in vivo assays has been slower than the adoption of SIAT1 for in vitro assays simply because of the large cost associated with ferret models. A better understanding of the infection parameter differences between the mouse and ferret models could ease the translation of results obtained in mice into predictions for the course and outcome of infection in ferrets and humans.

Recently, efforts have been made to model in-host influenza infection dynamics with a target cell limited model, using experimental data to validate the results [28]–[30], but the models have been limited to a single target cell population. Population heterogeneity has been accounted for in epidemiological models [31]–[34], where individuals become infected through primary contact with an infected individual, and heterogeneity is introduced by varying the contact rates between subpopulations. Due to the absence of an intermediate infection agent (i.e., virions) in these models, their results are of limited applicability to in-host infections, where the infection progresses from infected cells to healthy cells via the production and dispersal of infectious virions. Target cell heterogeneity has also been considered for within-host models of HIV [35], [36], hepatitis B [37], [38] and hepatitis C [39] and has provided an explanation for multiple phases of infection [35], [39] or different courses of disease progression [36]–[38]. However, these models are fairly complex, containing multiple compartments [36], [39] or assuming that target cell populations differ in most or all parameters [35], [37], [38], making analysis and interpretation difficult.

In this paper, we propose a mathematical model consisting of two distinct cell populations which differ only in their susceptibility to infection by a given viral strain and their rate of virus production. This choice is motivated by the assumption that differences in the cells' surface receptors will most directly affect the rate at which virus can bind cells to successfully infect them, as well as the rate at which newly produced virions will be able to break free from these cells after binding to their receptors upon budding. This simple model allows us to study the possible effects of cell tropism on the in-host progression and severity of influenza infections. By constraining one cell population to the specific parameter values defined in [28] for in-host human infection with influenza A (H1N1), we explore the dynamics of the model in the parameter space of the secondary cell population.

Unfortunately, to date, no publication has attempted to isolate a specific cell population's infection characteristics (e.g., viral production rate, cell infection rates) so it is not possible to use external data to set the value of the parameters relating to cell tropism in our model. Therefore, the work presented herein is an investigation of the effect cell tropism *could* have on infection dynamics and treatment strategies, rather than the effect it *will* have. We also do not attempt to differentiate between all possible causes for change in disease severity among different influenza strains. To our knowledge, there is not enough data available anywhere, and no data of the right nature (e.g., time course of IFN levels, Abs levels) to permit such a study.

The aim of this paper is two-fold: (1) to theoretically explore the parameter space of an infection model consisting in two target cell populations to understand what role, if any, cell tropism could play in modulating an influenza infection's dynamics; and (2) to consider what implications such an effect would have on treatment with antivirals. We find that the parameter space of the two target cell model contains a region of increased disease severity characterized by a larger viral titer peak and a long-lasting infection with high, sustained viral titer. We show that the long-lasting, sustained viral titer seen with more severe infections offers a longer window for effective treatment with neuraminidase inhibitors (NAIs) and adamantanes (or M2 blockers).

## Methods

### Mathematical model

The proposed two target cell model, which consists of two cell populations both susceptible to influenza virus infection, is an extension of the differential equation model consisting of a single susceptible cell population and delayed viral production proposed in [28], and fitted therein to match the dynamics of a primary influenza A/HK/123/77 (H1N1) infection in human volunteers. The two target cell model consists of a population of default (subscript 
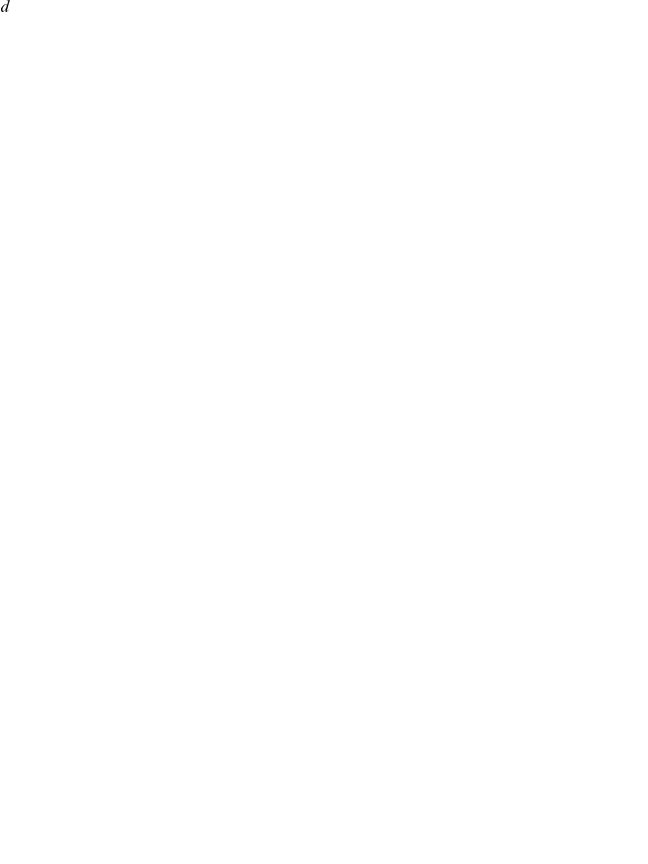
) and secondary (subscript 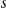) cells, namely
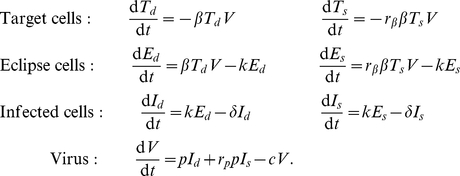
(1)Infection proceeds as target cells 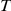 are infected by virus 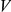 at a rate 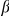 (or 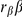). The newly infected cells 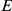 first enter a latent infection stage, called the eclipse phase, and turn into productively infected cells 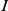 at a rate 
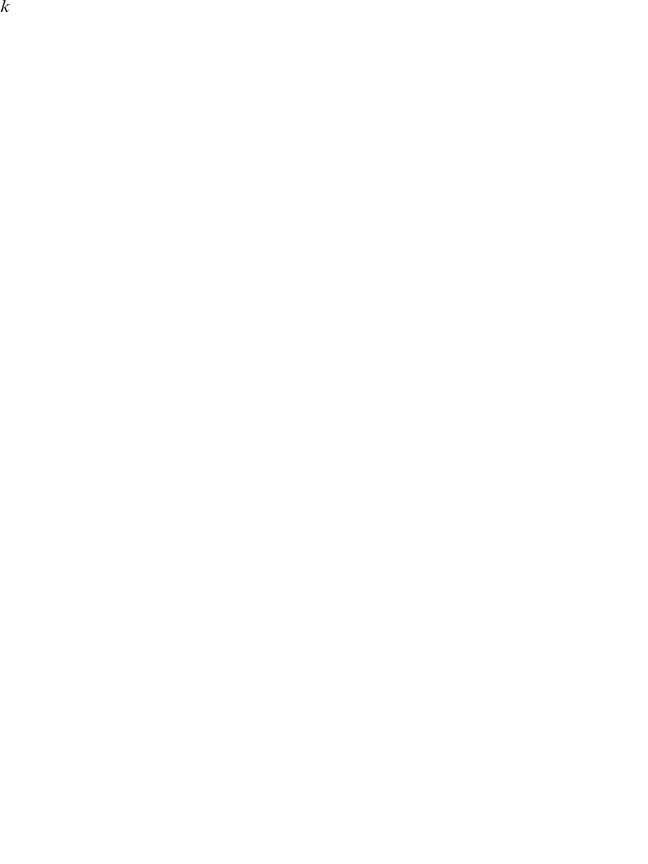
. Productively infected cells produce virus at a rate 
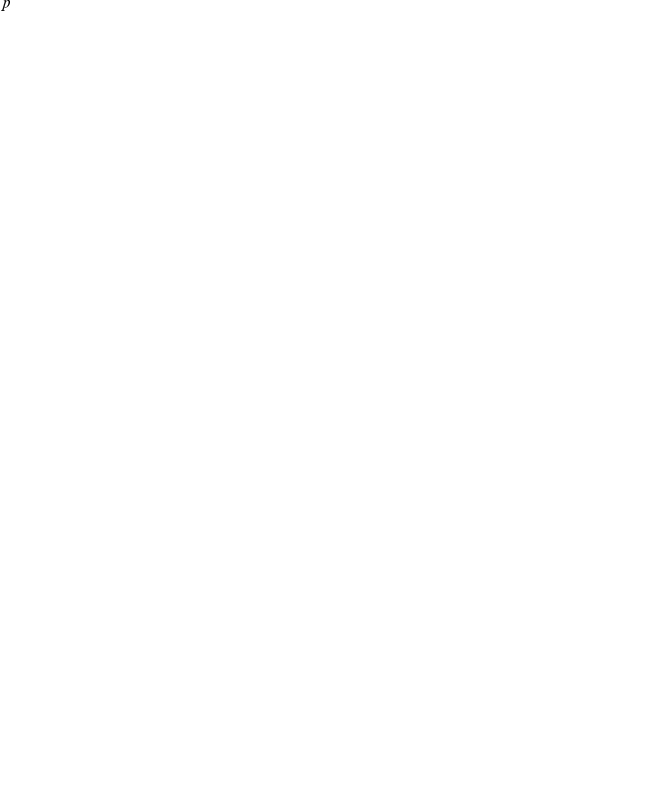
 (or 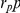) which is cleared at a rate 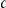 by the immune system or through loss of infectivity. The remaining virions go on to infect new target cells, and the infection progresses.

Differences between the two cell populations are controlled by three key parameters: 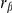, 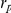, and 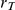. These parameters represent the fold difference in susceptibility to infection, 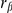, and viral production rate, 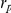, of the secondary cell type compared to that of the default cell type, and the fraction of cells of the secondary type in the initial target cell population, 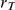. For example, setting 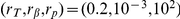 corresponds to a cell population where 20% of cells are of the secondary type and are 1,000-fold less susceptible to infection compared to cells of the default type, but once infected these secondary cells will produce 100-fold more virus than cells of the default type.

While 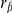 and 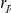 are scaling factors for parameters 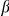 and 
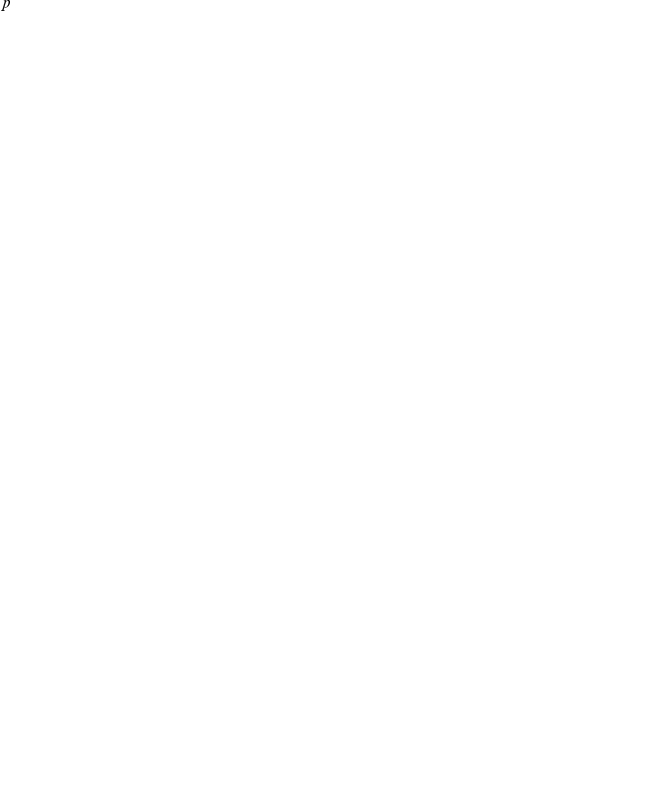
 in the secondary cells, respectively, 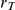 does not appear explicitly in the model as it is set through the initial conditions for target cells such that
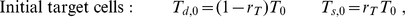
where 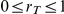.

The total number of initial target cells is set to 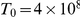 cells, which is in line with anatomical estimates for the human upper respiratory tract [28], and infection is initiated by a virus inoculum 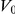. Default parameter values and initial conditions for (1) are listed in Table 1. These parameter values were obtained by repeating the individual fits to viral titers from six different volunteers infected with influenza A/Hong Kong/123/77 (H1N1) presented in [28]. We obtained different best-fit parameters which resulted in better SSRs than those presented in [28] which is why the geometric averaged best-fit parameters listed in Table 1 differ from those reported in [28] (parameters of the individual fits are available upon request). Numerical solutions of model (1) were obtained using the lsode function in Octave 3.0.1 [40], which uses an implementation of a backward differentiation formula (Gear's method) if the equations are stiff; otherwise, Adam's (predictor-corrector) method is used [41].

**Table 1:** Default initial conditions and parameter values of model (1).

Symbol	Parameter	Value
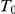	number of initially available target cells	4 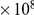 cells
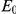 , 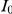	number of initially infected cells	0
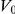	initial viral inoculum	7.6 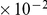 [V]^a^
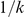	length of eclipse phase	4.2 h
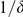	lifespan of productively infected cells	2.9 h
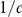	virus clearance rate	2.9 h
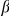	infection rate of cells by virus	1.0 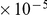 [V]^−1^·d^−1^
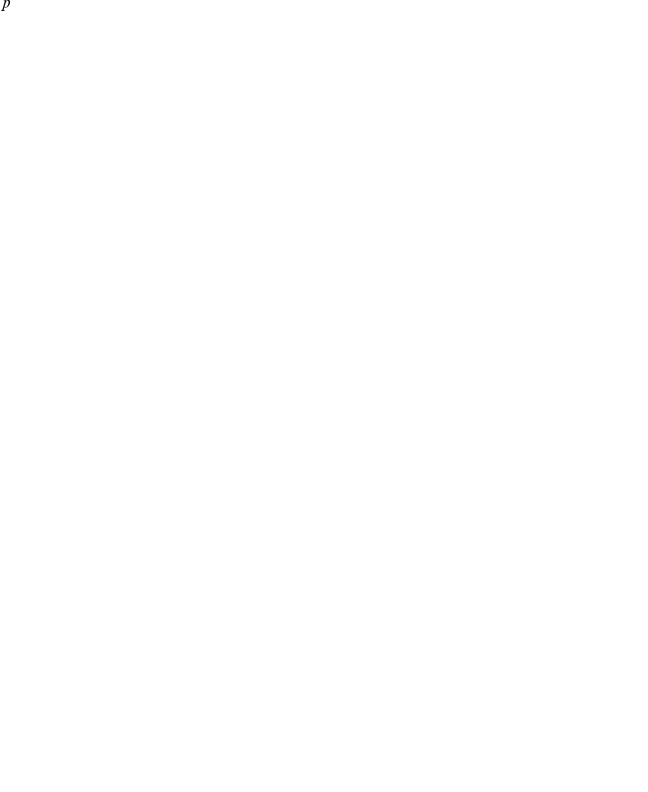	virus production rate	0.20 [V]·d^−1^
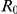	basic reproductive number	12

a Viral titer measured in units of 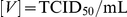 of nasal wash.

### Fits of the model to experimental data

To assess the validity and relevance of the two target cell model, we compared its performance to that of the single target cell model in capturing the dynamics of influenza infection of mice and humans. For fitting purposes, we define the default and secondary cell types as cells expressing either the SA
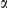
2,6 Gal or SA
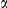
2,3 Gal receptors as described below.

In an effort to reduce the number of free parameters, we fix the number of total target cells based on measurements of the surface area of the human upper respiratory tract, 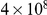 [28], or of the mouse lung, 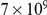 [42]. Note that the value used for the initial number of target cells will affect the values obtained for the best-fit viral production rates, but will not affect the ratio of virus produced by one cell population to the other. For the mouse model, we fix the proportion of cells predominantly expressing SA
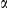
2,6 Gal receptors on their surface, 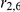, at 10%. This is an approximate value based solely on qualitative reports indicating that mice lung cells predominantly express SA
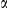
2,3 Gal receptors [25]–[27]. For the human model, we fix the proportion of cells predominantly expressing SA
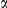
2,6 Gal receptors on their surface, 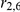, at 70%. This is based on studies of human lung physiology indicating that the epithelium of the upper airway (up to the fifth generation) comprises 50–85% nonciliated cells [43], and reports indicating that nonciliated cells predominantly express SA
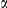
2,6 Gal receptors on their surface [20], [22], [23].

Setting an exact value for the fraction of cells predominantly expressing SA
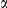
2,6 Gal receptors on their surface, 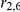, is not essential since a change in 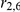 can be corrected by appropriate re-scaling of 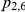 and 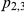. That is, if 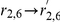 then we have
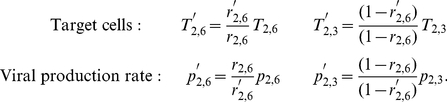
(2)All other parameters were determined by fitting 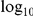 of the viral titer predicted by the model to that of the experimental data. The fits were performed using either the Octave 3.0.1 [40] leasqr function, which is an implementation of the Levenberg-Marquardt nonlinear regression method [44], or the nelder_mead_min function, which uses the Nelder-Mead method for finding the minimum of a function. The fits presented here are the best fits found using either one of these methods. For comparison, we also fit the data using the single target cell eclipse model [28].

To quantify the quality of each fit, we computed the sum of squared residuals (SSR) between the experimental viral titer and the models' results. In order to compare models with different numbers of parameters, we also computed small-sample size (second order) Akaike's “an information criterion” (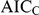). The model with the lowest 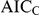 is considered to be best supported by the experimental data available.

## Results

### Mapping the parameter space

Since differences in cell receptor specificity between influenza A strains of human and avian origin, and changes in viral production for different cell types have not been quantified [16], [45], [46], we consider a wide range of parameter values for the secondary cell population. The susceptibility to infection of target cells of the secondary type, 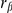, and their rate of virus production once infected, 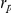, are independently varied from 1,000-fold less (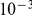) to 1,000-fold more (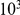) than that of the default cell type.

**Figure 1:**
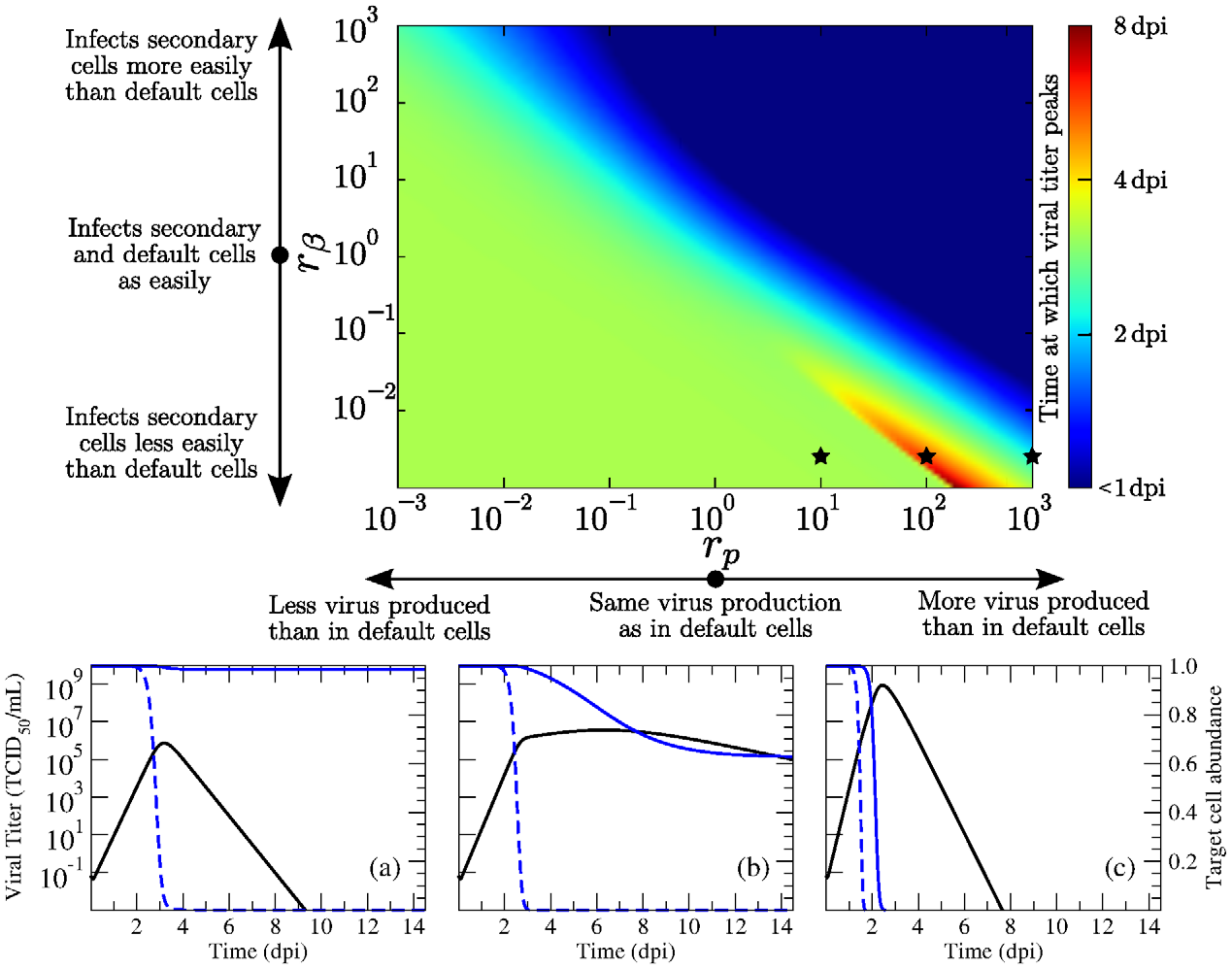
Time of viral titer peak for different properties of the secondary cell type. The effect of varying the secondary cells' susceptibility to infection (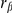) and their rate of virus production (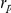) relative to that of the default cell type is illustrated (top) for a population of cells with an equal abundance of default and secondary cells (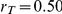). Time of viral titer peak is given in days post-infection (see legend to the right of graph). The three stars indicate the specific parameter values used in the three graphs (bottom) from left to right (a to c). The graphs depict the viral titer (black), and the relative abundance of target cells of the default (blue, dashed) and secondary (blue, solid) type over the course of an in-host influenza infection when the secondary target cells' susceptibility to infection is 500-fold less, and their virus production rate is (a) 10-, (b) 100-, and (c) 1,000-fold higher than that for cells of the default type. All other parameter values are given in Table 1.

Figure 1 illustrates how the time post-infection at which viral titer peaks depends on the fold difference in susceptibility to infection (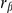) and viral production rate (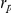) of the secondary cell type compared to that of the default cell type. Here we present only the case where default and secondary target cells are present in equal numbers (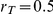). Varying the fraction of target cells of the secondary type results in similar behaviour, except in the case of a nearly homogeneous cell population composed mainly of cells of the secondary type (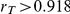).

When 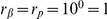, the two target cell model reduces to the single target cell model and the viral titer curve reaches a peak value around 3 days post-infection (dpi), as with the single target cell model [28]. Not surprisingly, increasing the secondary cells' susceptibility to infection (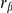) or their rate of virus production (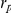) relative to that of the default cell type causes the infection to peak earlier. This is because with a large 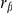, cells of the secondary type are infected more easily and therefore consumed more rapidly by the infection leading to a shorter-lasting infection. Analogously, with a large 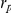, secondary cells release larger amounts of virus once infected, which in turn leads to a more rapid consumption of all cell types by the infection. This translates to a shorter-lasting infection as one moves upwards or rightwards on the graph in Figure 1, with the shortest-lasting infections found in the top right corner of the graph.

In general, over the parameter space explored, the viral titer peaks between 1 dpi and 4 dpi, with the exception of an unexpected pocket in the parameter space at the bottom-right of our graph in the region where cells of the secondary type produce more virus than cells of the default type (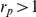), but are harder to infect (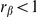). In the vicinity of this pocket, the time of viral peak varies rapidly from 2 dpi to more than 8 dpi, becoming increasingly sensitive to the secondary cells' susceptibility to infection and their viral production rate. Within this parameter region, secondary cells are not easily infected, due to their low susceptibility to infection (small 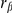), and as such these cells are consumed very slowly by the infection. On the other hand, their high rate of virus production (large 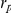) means that once infected, even in very small numbers, these cells produce large quantities of virus. As a result, the viral titer is sustained at high levels long after the onset of infection, and peaks substantially later than in other regions of the parameter space.

This is well illustrated in the three examples presented in Figure 1(a)–(c) where the kinetics of the infection are shown for three different viral production rates of the secondary cell population for the case where these cells are 500-fold harder to infect than cells of the default type. When secondary cells produce only 10-fold more virus than cells of the default type, the infection is mostly limited to the default cell population as the amount of virus produced is not sufficient for the infection to spread to the secondary cell population. Increasing the production rate to 100-fold more than cells of the default type results in a sufficient amount of virus being produced to sustain a slow growing infection within the secondary cell population, leading to long-lasting, high-levels of viral titer. Finally, increasing the viral production rate to 1,000-fold more than the default cell type allows the infection to successfully consume both cell populations rapidly.

It is important to note that while the secondary cells must be harder to infect than the default cells, the secondary cells do not require a higher viral production rate to achieve this sustained viral titer. The same kinetics can be achieved with secondary cells having a similar or even a lower viral production rate than default cells if the ratio of default to secondary cells is adjusted according to equations (2). The specific conditions for parameters that lead to sustained viral titer are discussed further below.

### Conditions for infection

Figure 1 suggests that the two target cell model leads to high, sustained, viral titers over a certain region of the parameter space. In order to better understand the relationship that needs to exist between the secondary cell population's susceptibility to infection and viral production rate to give rise to a severe, long-lasting infection, it is useful to consider a linear stability analysis of the two target cell model. For all parameter values, the equations have a line of fixed points, corresponding to a stable, uninfected cell population persisting in the absence of infection, namely 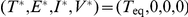, where 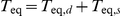. Within each the default and secondary cell types, this equilibrium value is less than or equal to the initial cell population, i.e., 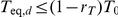 and 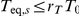. Therefore, the stability of a fixed point is guaranteed by the condition 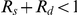, where
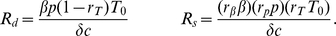
Thus, when 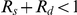, an initial quantity of virus does not lead to a substantial infection of the cell population, and growth of the virus is suppressed. For a single target cell population, i.e., 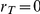 or 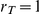, the stability conditions are 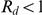 or 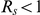, respectively.

The three quantities, 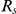, 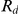 and their sum, are analogous to the basic reproductive number 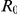, a frequently used quantity in epidemiology and in-host infection dynamics, which is defined as the average number of second-generation infections produced by a single infected cell within a homogeneous population of completely susceptible cells [47], [48]. Although this description of 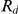 and 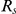 may be valid in the case of a homogeneous cell population, the interpretation for a heterogeneous population is less straightforward.

**Figure 2:**
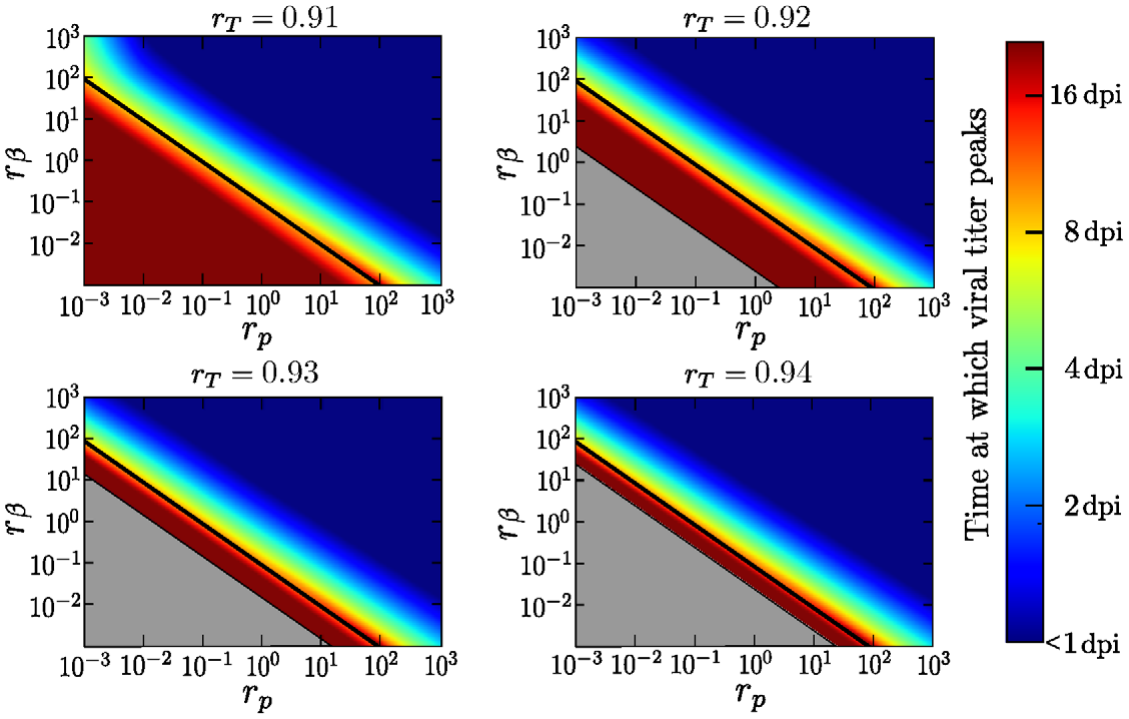
Time of viral load peak when the cell population is mostly composed of secondary cells. Different graphs represent different proportion of secondary target cells, 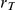, for values ranging from 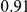
–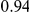. Each graph explores the effect of varying the secondary cells' susceptibility to infection (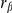) and their rate of virus production (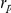) relative to that of the default cell type. All other parameters are held fixed at the values presented in Table 1. The stability condition 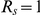 (black line) is indicated. Grey regions indicate regions where the infection fails to spread to either cell population (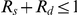). Time of viral titer peak is given in days post-infection (see legend on right side of graphs).

For the parameter values presented in Table 1, the quantity 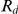 is always greater than one when 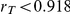, resulting in growth of the viral titer. This results from the choice of default parameter values, which were taken from an infection where 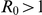. When 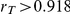, regions arise within the parameter space where 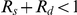; that is, the initial viral titer fails to lead to an infection. We also see a region where the individual quantities 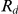 and 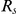 are both less than one, but the sum of these quantities is greater than one. This implies that the infection grows slowly, and although not explicitly accounted for in our model, the host immune response would likely intervene before the viral titer reached symptomatic levels. These cases are illustrated in Figure 2.

In order to narrow our focus to biologically relevant regions of the parameter space, we will restrict our analysis to the case where 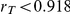. The quantity 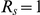 will be seen to form a boundary in the parameter space which establishes a region leading to high viral loads which are sustained for long periods of time.

### Implications for disease severity

Beyond simply mapping the infection kinetics through the parameter space, it is important to understand the implication of a given viral titer curve on the severity of infection for a patient. To this end, we introduce three approximate measures of viral infection severity: time of viral load peak, approximate duration of symptomatic infection, and total amount of virus. Each measure has its advantages and limitations, but together they provide a better understanding of the infection dynamics in terms of how it may be experienced by a patient. Other possible measures of disease severity which may be more difficult to estimate clinically are presented in Appendix S1.

In order to focus on the specific parameter region which results in severe, long-lasting infections characterized by high-level, sustained viral loads, we restrict our analysis to the region of the parameter space bounded by 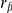, the relative susceptibility of secondary cells to infection, from 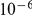 to 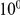, and 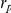, the relative viral production rate by secondary cells, from 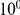 to 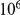.

#### Time of viral titer peak

**Figure 3:**
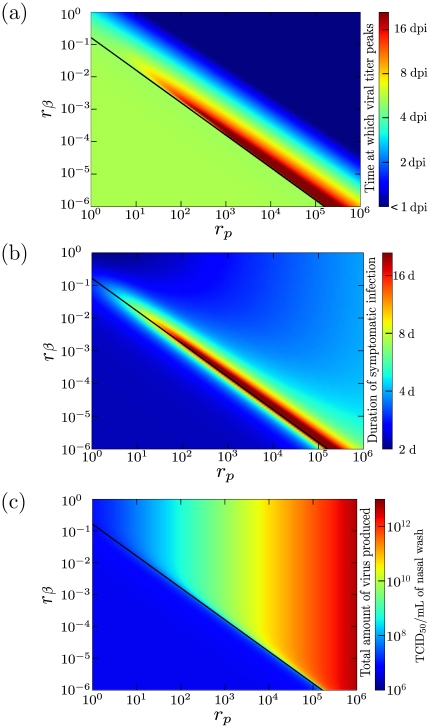
Measures of disease severity for varying properties of the secondary cell population. The effect of varying the secondary cell's susceptibility to infection (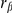) and their rate of virus production (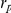) compared to that for cells of the default type on different measures of disease severity: (a) time of viral titer peak, (b) duration of symptomatic infection and (c) total amount of virus produced. We have adjusted the axes to examine the region of sustained viral titer. Note that the value of the disease severity measures in the top left corner of each graph (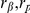 = 1,1) corresponds to the single target cell model. All other parameters are set as specified in Table 1, with 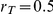. The stability condition 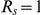 is also indicated (black line).

A useful measure of disease severity is the time at which the viral load reaches its maximum value. This is an important measure because, as seen in [28], when treatment with a neuraminidase inhibitor such as oseltamivir is applied after viral titer peak, it has little effect on disease severity and duration. Thus, the time of viral titer peak provides an estimate of the time window available for effective treatment. In general, an early viral titer peak means that both external treatment or host immunity has little opportunity to act to reduce disease morbidity. The dependence of the time of viral titer peak on the secondary cells' infection characteristics are shown in Figure 3(a).

As the secondary cells' susceptibility to infection (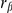) and their viral productivity (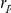) decrease, the viral titer peaks progressively later, going from 1 dpi to as late as 16 dpi. In the region where 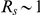, the late peak of viral titer is due to the slow consumption of the secondary cells. This late viral titer peak means that even relatively late treatment with antivirals could have a noticeable, beneficial effect in reducing disease morbidity and perhaps avoiding mortality in such infections. We will return to this below when we simulate treatment of these infections with neuraminidase inhibitors and adamantanes.

#### Symptomatic infection duration

Though our model predicts that viral titer can peak significantly later for certain parameter regimes, this does not necessarily imply a sustained, severe infection. For example, it is important to distinguish between a small, slow growing infection, and one which grows rapidly and is maintained over a long period of time, with a late viral titer peak. While the former would likely be cleared effectively by an immune response before it has the chance to fully develop, the latter might already be too severe by the time the immune response gets underway, resulting in a severe infection rendered more morbid by the extensive immune response triggered by the high and long-lasting viral titer. Thus, we establish another measure of infection severity, which we define as the duration of the symptomatic infection. The dynamical markers for disease severity are not well known, but based on patient symptom scores, the onset of symptoms in a human-derived influenza infection appears to take place sometime between 1–2 dpi, and to dissipate around 5–6 dpi [49], [50]. On the viral titer curves for human-derived strain infection, these two time points correspond approximately to places where the viral titer curve crosses 10^4^ TCID_50_/mL (see Figure 1(a)). Thus, we set this viral titer as the threshold level corresponding approximately to that required for a symptomatic infection. Following this convention, we define the duration of the symptomatic infection to be the length of time for which the viral titer remains above the symptomatic threshold. This is shown in Figure 3(b).

We find that the region corresponding to a late peak of the viral load in Figure 3(a) also corresponds to a viral load sustained above the symptomatic threshold long after the onset of infection. The viral titer surpasses the symptomatic threshold at approximately 2 dpi, as we can see in Figure 1(b). We can therefore exclude the possibility that the late peak of the viral titer results from the slow and steady growth of the viral titer. Rather, the late peak is the result of sustained viral titer at high levels. This behaviour can be explained by considering the infection of the two populations separately. After the default cell population is completely consumed by the infection, approximately 3 dpi, an essentially homogeneous population of secondary cells remains. The quantity 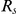 is then analogous to the basic reproductive number for the secondary cell population. Thus, when 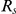 is slightly greater than one, growth of viral titer occurs slowly, and the viral titer is sustained at the high levels produced during infection of the default cell population, as illustrated in Figure 1(b). This also has implications for treatment strategies. While a long lasting treatment regimen usually makes little sense for treating seasonal influenza infections, it would be beneficial if not necessary for controlling a longer-lasting infection characterized by sustained viral production.

#### Total virus produced

In assessing infection severity, it is also helpful to consider the total amount of virus produced over the course of the infection. Severe influenza viral infections are characterized by high viral loads [51], which does not necessarily follow from a delayed peak of the viral titer or a long symptomatic infection. The total amount of virus produced during the course of infection was determined using
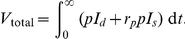
The results within the parameter space are shown in Figure 3(c). When 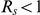, the total amount of virus produced is independent of 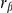 and 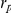 as the viral titer does not grow to sufficient levels to establish an infection within the secondary cell population. When 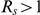, there are sufficiently large rates of virus production and target cell infectivity for the infection to spread within the secondary cell population. Figure 3(c) illustrates that when 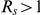, the amount of virus produced is predominantly dependent on the scaling factor for the rate of virus production, 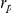. When 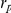 is large, a significant amount of viral titer is produced and a large number of cells from both populations are consumed, regardless of the secondary cells' susceptibility to infection. However, as 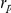 decreases, the quantity 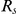 approaches one, and the presence of long-lasting infection becomes increasingly sensitive to variations of either 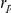 or 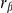.

This feature is particularly interesting when framed within the context of anti-influenza drug treatment. For infection characterized by 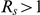, depending on the value of 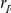 and 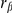, i.e. where you are in the parameter space, it is sometimes preferable to treat with an antiviral targeting the secondary cells' susceptibility to infection, 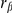, such as an adamantane so as to move downwards in the parameter space to most easily reach 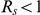. In other cases, it is more beneficial for an equivalent drug efficacy to treat with an antiviral targeting the secondary cells' viral production rate, 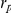, such as a neuraminidase inhibitor so as to move leftwards in the parameter space to most easily reach 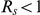.

Note that the plot for total virus produced as a function of 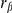 and 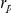 is identical in feature to that of another important measure: the total amount of free virus which is defined as the area under the curve (AUC) of free virus (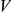) over time (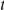). The total amount of free virus is important as it is related to the epidemiological (host-to-host) transmission fitness of a particular strain [30].

### Fits of the model to experimental data

We have seen that the two target cell model is capable of producing high, sustained, viral titers. To determine whether these sustained viral titers are representative of the dynamics observed for severe influenza infections in vivo, and to compare our two target cell model's performance to that of the single target cell model, we fitted both models to measurements of influenza infections in mice [52] and humans [7].

From here on, we will refer to the two target cell populations as those predominantly expressing SA
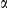
2,6 Gal or SA
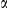
2,3 Gal surface receptors rather than as “default” and “secondary” cells. Accordingly, we replace parameters 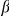, 
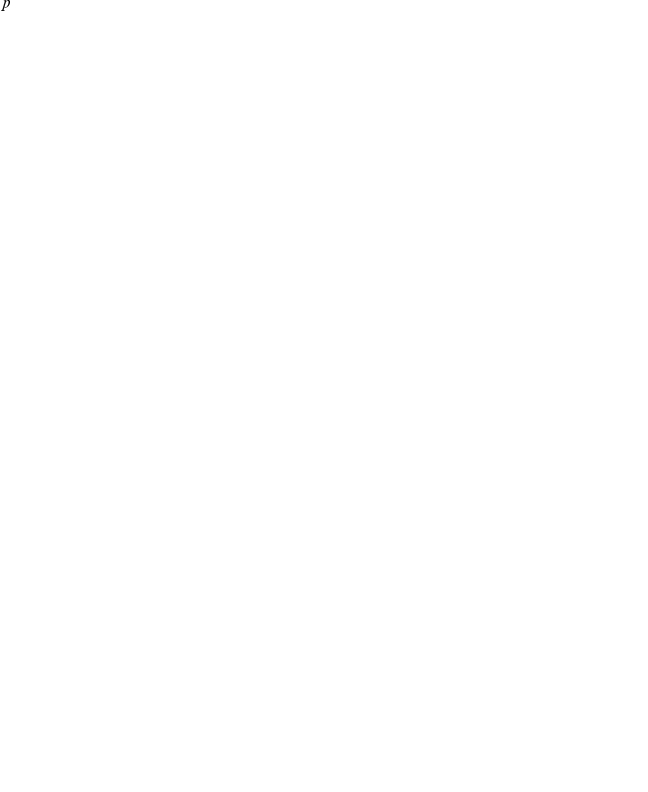
, 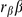, and 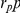, with parameters 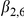 and 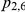, the infection and production rates associated with cells predominantly expressing SA
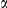
2,6 Gal receptors on their surface, and 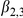 and 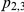, the infection and production rates associated with cells predominantly expressing SA
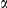
2,3 Gal receptors on their surface, which are easier to interpret in terms of what they represent biologically.

#### Influenza infection in mice

In the first data set, BALB/c mice were infected intranasally with 10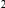 pfu of A/Texas/36/91 (H1N1), A/1918 (H1N1), A/Thailand/SP/83/2004 (H5N1), or A/Thailand/16/2004 (H5N1) influenza virus. Lungs from 3 mice were harvested and homogenized at 1, 3, 4, 5, and 7 dpi. Virus titers were determined by plaque assay in MDCK cells. Although two of the strains are avian-derived and the other two are human-derived, all four strains produced infections with high, sustained, viral titers. This is surprising given that mice lung cells predominantly express SA
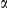
2,3 Gal receptors on their surface [25]–[27] and as such one would expect that strains which prefer SA
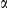
2,3 Gal receptors (e.g., H5N1) would replicate more effectively in mice than those which prefer SA
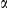
2,6 Gal receptors (e.g., H1N1).

**Figure 4:**
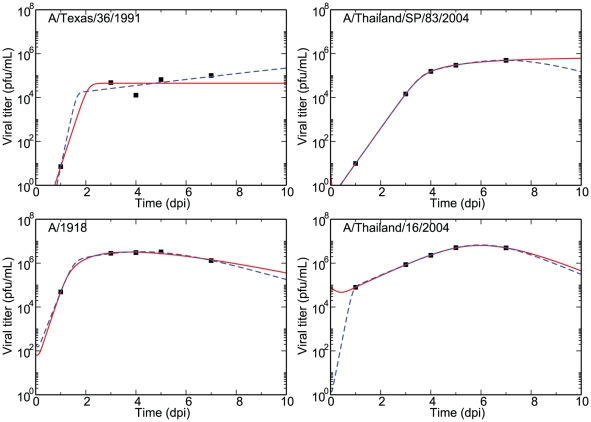
Model parameter fits to experimental influenza infection in mice. Results of parameter fits of the single target cell (solid line) and two target cell (dashed line) eclipse model to human-strain (A/Texas/36/1991 and A/1918) and avian-strain (A/Thai/SP/83/2004 and A/Thai/16/2004) influenza infections in mice. The percentage of cells expressing the SA
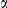
2,6 Gal receptor, 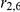, is fixed to 10%, and the best fit parameters are presented in Table 2. For the single target cell model, the SSRs are (from left to right and top to bottom) 0.45, 2.8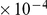, 7.8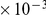, and 3.3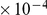 while the 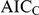 are −35, −72, −56, and −71. For the two target cell model, the SSRs are 0.29, 2.1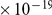, 2.3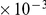, and 7.4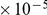 while the 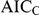 are −32, −240, −56, and −74. Data is taken from [52].

Figure 4 shows our model fits to influenza infections in mice, with the solid line indicating the best fit for the single target cell model and the dashed line indicating the best fit for the two target cell model. Parameters for the best fits are given in Table 2. Visually, it appears that both models can adequately capture the dynamics of both human- and avian-strain influenza infections in mice. While the SSR are always smaller for the two target cell models because it has two additional free parameters, the 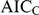 are comparable between the two models suggesting that the two target cell model is equally well supported by the experimental data despite its additional parameters.

**Table 2:** Model parameter fits for experimental influenza infection in mice.

	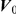 ([V])	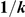 (h)	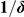 (h)	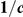 (h)	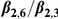 ( 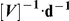 )	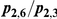 ( 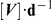 )	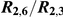	SSR	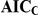
Single target cell model									
LH	5.9 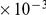	4.4 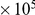	7.3	1.2	1.1 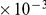 /−	7.7/−	8.8 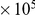 /−	0.45	−35
LA	8.2	240	130	0.41	6.9 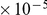 /−	0.019/−	810/−	2.8 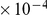	−72
HH	65	33	32	34	9.8 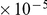 /−	0.0012/−	1600/−	7.8 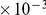	−56
HA	1.5 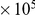	16	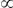	7.1	5.9 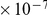 /−	0.0027/−	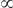 /−	3.3 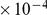	−71
Two target cell model ( 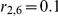 )									
LH	4.1 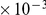	24	0.74	0.80	2.4 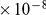 /8.2 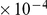	77/0.0030	1.3/16	0.29	−32
LA	1.4	9.2	1.1	1.3	2.7 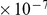 /3.3 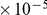	2.9/0.0039	1.3/2.0	2.1 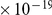	−240
HH	19	4.9	5.0	5.0	5.2 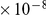 /2.4 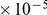	0.90/0.0033	1.4/22	2.3 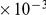	−56
HA	4.8 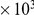	2.9	3.5	3.8	4.1 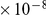 /9.4 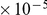	2.4/2.2 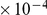	1.6/3.0	7.4 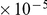	−74

LH: Low-pathogenic human A/Texas/36/91 (H1N1).

HH: High-pathogenic human A/1918 (H1N1).

LA: Low-pathogenic avian A/Thai/SP/83/2004 (H5N1).

HA: High-pathogenic avian A/Thai/16/2004 (H5N1).

It is, however, also important to consider whether the parameter values of the best fits are biologically realistic. The basic reproductive numbers, 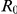, obtained with the single target cell model are rather large (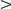
810) compared to those obtained for with the two target cell model (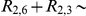
3–23) which are more in line with values obtained typically (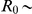
3–75) for infections with human strains [28], [29]. In addition, while the values obtained for the eclipse delay (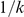) and infected cell lifespan (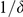) are unrealistically large for the fits of the single target cell model, these values are typically reasonable for the two target cell model fits. We have also tried fixing the unrealistic parameters (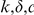) to the base values from Table 1 and found that under this constraint, the single target cell model does poorly (SSR 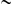 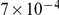
−2) compared to the two target cell model (SSR 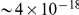
−0.3, results not shown).

Unfortunately, interpretation of the cell tropism of these four strains from their parameter values for the two target cell model fits is awkward. All four strains display a preference for cells expressing SA
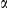
2,3 Gal over those expressing SA
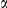
2,6 Gal (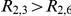), with human strains exhibiting a stronger preference for cells expressing SA
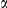
2,3 Gal than avian strains. This is not consistent with expectations that the two human-adapted H1N1 strains should prefer cells expressing SA
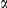
2,6 Gal while the avian-derived H5N1 strains should prefer cells expressing SA
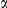
2,3 Gal. This could be a consequence of the overparametrization (8 parameters for 5 data points). The set of best fit parameters presented here is part of a larger family of best fit parameters sets which capture this data equally well (see for example the rescaling of 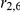, 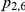 discussed in the Methods section), and which likely contains more reasonable cell tropism parameters which would fit the data equally well. If some of the parameters of the two target cell model could be resolved through experiments, it would restrict the set of possible parameter values which could help validate or invalidate the two target cell model.

Therefore, while the single- and two-target cell models each provide good fits which are well supported by the data, the two target cell model does so under more realistic parameter values, suggesting that it offers a better description of the dynamics. However, this likely says more about the limitations of the single target cell model than about the appropriateness of the two target cell model. Indeed, one can imagine that other extensions of the single target cell model (e.g., by including an immune response) would do equally well if not better than the two target cell model.

#### Influenza infection in humans

The second data set consists of pharyngeal and nasal swabs collected from 18 patients infected with avian (H5N1) influenza, and 6 patients infected with human influenza (either H1N1 or H3N2) upon admission to hospital. This data set shows a clear dynamical difference between human- and avian-adapted influenza strains, with avian-strain infections peaking later and lasting longer than infections with human-adapted influenza strains.

**Figure 5:**
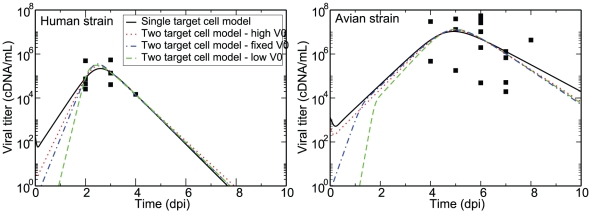
Model parameter fits of human and avian influenza infections in humans. Results of parameter fits of natural human infections with either a human (left) or avian (right) influenza strain. The data was fit using either the single target cell (solid) or the two target cell model where the fits were initialized with a guess for the virus inoculum which was either high (dotted), low (dashed), or fixed at 1 cDNA (dash-dot). The percentage of cells expressing the SA
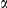
2,6 Gal receptor, 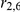, is fixed to 70%, and the best fit parameters are presented in Table 3. Data is taken from [7].

Figure 5 shows several fits of the single- and two-target cell models to data from natural human- and avian-strain influenza infections in humans with the resulting parameters given in Table 3. The solid line uses the single target cell model and the remaining lines use the two target cell model. While the SSR is comparable for the fits of the single- and two-target cell models, the lower 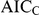 for the fit of the single target cell model to the avian strain infections suggests that it may be better supported than the two target cell model by the limited data available.

**Table 3:** Model parameter fits for influenza infection in humans.

	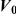 ([V])	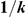 (h)	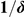 (h)	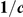 (h)	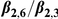 ( 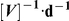 )	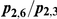 ( 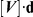 )	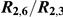	SSR	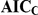
Single target cell model									
H	180	7.3	1.2	1.4	1.2 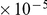 /−	0.25/−	3.2/−	1.6	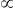
A	1300	16	4.5	3.4	2.4 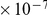 /−	2.0/−	5.3/−	22	29
Two target cell model ( 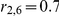 )									
HH	8.4	2.3	2.3	0.94	1.6 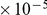 /1.8 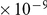	0.14/600	2.4/0.5	1.6	−85
AH	590	11	1.6	2.2	1.2 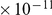 /1.4 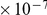	3100/31	0.061/3.1	21	44
HL	9.1 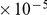	6.2	1.1	9.3	5.2 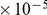 /1.6 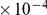	0.036/0.061	13/69	1.6	−85
AL	5.0 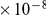	11	1.4	1.1	1.2 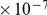 /4.5 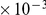	34/0.015	3.0/20	21	44
HF	(1.0)	3.3	4.4	3.6	3.8 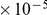 /2.5 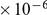	0.22/43	7.3/0.48	1.6	−141
AF	(1.0)	1.9	0.78	9.0	9.6 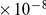 /2.0 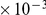	7.6/0.0018	2.5/5.0	22	35

H,A: Fits to the human and avian data sets, respectively.

H,L,F: Indicates whether the initial guess for the viral titer was high, low, or fixed, respectively.

Fits of the single target cell model yielded mostly reasonable parameter values with the increased severity of avian-derived strains compared to human strains reflected in the larger 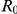 value (5.3 vs 3.2). The parameter fits of the single target cell model suggest that compared to human-derived strains, the avian strains infect cells less effectively (smaller 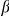), but once infected, cells produce more virus (larger 
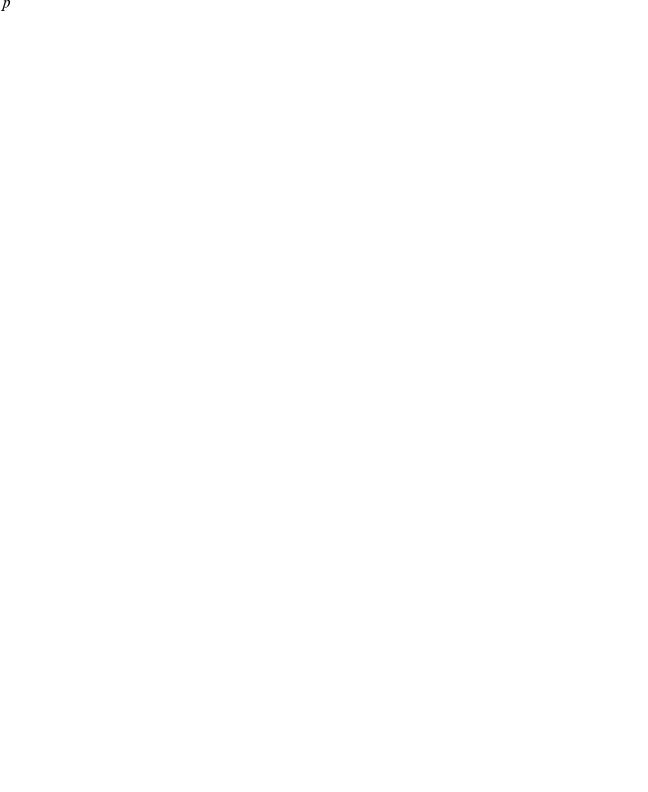
). In addition, the longer infected cell lifespan (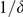) and smaller viral clearance rate (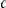) for avian strains could be the result of a less effective immune response to the novel, avian-derived strains. However, the data is too limited to lend any weight to these interpretations.

For fits of the two target cell model, the best fit parameters, especially those characterizing cell tropism, depend on the value used as the guess for the initial viral inoculum, 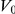, when initializing the fitting programme. When initialized with a high value for 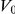 (dotted line in Figure 5), the solver converges to fits which reduce the two target cell model to a single target cell model (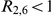 or 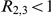) for both human- and avian-strain infections. When initialized with a low value for 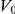 (dashed line in Figure 5), the solver converges to fits in which both cell populations participate in the infection for both human- and avian-strain infections.

Unlike the fits to the mice data, fits to the human data give reasonable parameter values for both models. The key difference between the fits of the single and two target cell models is that the former appears to require larger initial viral inoculum to fit the infections with avian strains. This is because the sustained viral titer suggested by the data can only be captured by the single target cell model when infection proceeds slowly through the only cell population available, allowing the infection to be sustained. In turn, the slow infection growth requires that viral titer be high from the start to match the high levels seen in the data. It is possible that the single target cell model is correct and that influenza infections with avian-derived strains are actually initiated with larger initial inoculum compared to seasonal infections with human-adapted strains. For example, this could be a consequence of the former being contracted directly through contact with infected animals which could result in larger initial inoculum compared to seasonal infections which are most likely contracted though airborne particulates.

However, it is possible that the difference in inoculum between infections with human- vs avian-derived strains is merely a consequence of the dynamical limitations of the single target cell model, and that in fact both natural infections are initiated with similar inoculum. To explore this possibility, we also present the result of fits of the two target cell model where the initial inoculum is fixed to an intermediate value (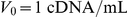, dash-dot line in Figure 5) for both infections with human and avian strains. The resulting best fit is essentially a single target cell model for human-strain influenza and a two target cell model for the avian-strain infections.

Regardless of the initial inoculum, fits with the two target cell model suggest that human strains infect cells expressing the SA
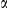
2,6 Gal receptor on their surface more easily than those expressing the SA
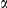
2,3 Gal receptor (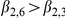). In contrast, avian strains infect cells expressing the SA
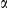
2,3 Gal receptor more easily (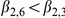). This is in line with what we know of these strains' preferences, with human strains preferring cells expressing SA
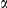
2,6 Gal receptors and avian strains preferring those expressing SA
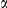
2,3 Gal receptors. More quantitative information such as measurements of the infection rates of each strain in each cell population would be required to properly calibrate the model and assess the validity of its predictions regarding, for example, the variations in the production rates of virus between cell types and viral strains.

In the end, as indicated by the 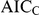 and SSR, the single target cell model is statistically the better explanation for both human- and avian-strain influenza as there is simply not enough data to support the extra parameters of the two target cell model. Thus, while the experimental data does not reject the possibility of cell tropism playing a role in driving the dynamical difference between infections with human versus avian influenza strains, it cannot confirm it either.

### Drug treatment in the two target cell model

Having extracted parameter values which can capture the dynamics of infections with human or avian influenza strains in humans enables us to explore how the predictions of the two models differ with respect to the efficacy of treatment with antivirals. Specifically, we compare the effect of treatment with neuraminidase inhibitors (NAIs), which block the release of viral particles, and adamantanes, which prevent uncoating of the virus, on human- and avian-strain influenza infections modelled with either a single target cell model (solid line of Figure 5) or a two target cell model (dashed line of Figure 5). The effect of NAIs is modelled as a reduction in the virus production rates, 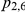 and 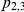, by multiplying them by 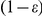, where 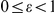 is the efficacy of the drug. Adamantanes are modelled in a similar fashion except that the effect of the drug is to reduce 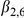 and 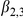 when they appear in the equation for 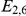 or 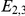, a formulation suggested by Beauchemin et al. [29]. We set 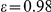 [28], [30] and examine the effects of prophylactic treatment, as well as treatment initiated at 2, 3, and 6 dpi.

**Figure 6:**
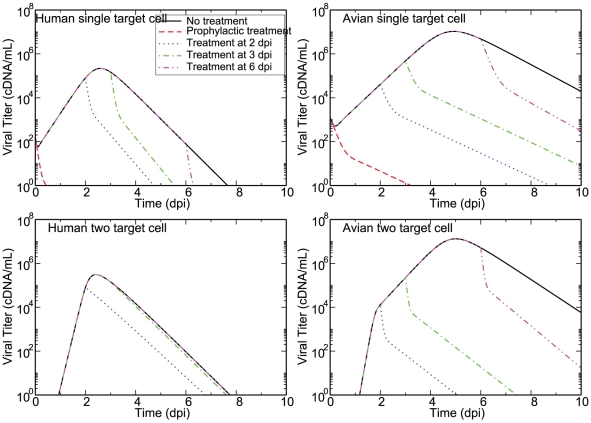
NAI treatment of human infections with either human or avian influenza strains. Viral titer over the course of infections with either a human or avian influenza strain modelled with either a two target cell model (low 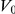, dashed line of Figure 5) or a single target cell model (solid line of Figure 5) in the presence of drug treatment is shown. NAIs are applied either prophylactically (dashed line), at 2 dpi (dotted line), at 3 dpi (dash-dot line), or at 6 dpi (dash-dot-dot line) with an efficacy of 0.98. Prophylactic treatment lines cannot be seen in the case of the two target cell model since the initial viral inoculum is below the range of the graph and prophylactic treatment suppresses the infection.

**Figure 7:**
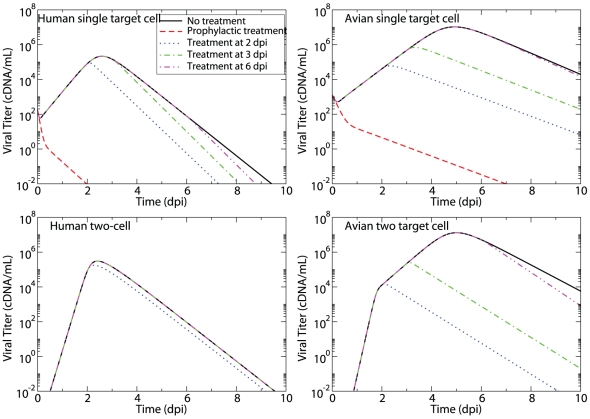
Adamantane treatment of human infections with either human or avian influenza strains. Viral titer over the course of infections with either a human or avian influenza strain modelled with either a two target cell model (low 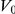, dashed line of Figure 5) or a single target cell model (solid line of Figure 5) in the presence of drug treatment is shown. Adamantanes are applied either prophylactically (dashed line), at 2 dpi (dotted line), at 3 dpi (dash-dot line), or at 6 dpi (dash-dot-dot line) with an efficacy of 0.98. Prophylactic treatment lines cannot be seen in the case of the two target cell model since the initial viral inoculum is below the range of the graph and prophylactic treatment suppresses the infection.

Figure 6 shows the effect of NAI treatment on viral titers over the course of human infections with either a human (left column) or an avian (right column) influenza strain modelled using either the single target cell model (top row) or the two target cell model (bottom row). For infections with human influenza strains, the predictions made by the single target cell model suggest that antivirals will be more effective than predicted by the two target cell model. This is because for the particular parameters used here for the single and two target cell models for infections with human-derived strains, infection in the two target cell model grows and peaks more rapidly, making delayed treatment interventions less effective. For infections with avian influenza strains, the predictions made by the single and two target cell models are similar, although the two target cell model predicts that treatment will be slightly more effective than predicted by the single target cell model. That is because in this case, it is the infection in the single target cell model which grows and peaks more rapidly, making delayed treatment less effective. Figure 7 shows the effect of adamantane treatment on the same human and avian infections. The results are similar to what is seen for NAIs although adamantanes do not reduce the duration of the infection as much as NAIs. The administration of NAIs initially causes a rapid drop in viral titer which leads to a quicker resolution of the infection, an effect that is not seen when adamantanes are administered.

Ultimately, these four simulations show that what determines the effectiveness of delayed treatment with antivirals is simply the growth rate of the infection and the time of viral titer peak. Infections which proceed slower and peak later provide an increased window of opportunity in which to administer antiviral treatment. In contrast, a fast infection which peaks early is not a good candidate for antiviral treatment because when administered on or after viral titer peak antivirals have little or no effect on reducing the severity and duration of the infection. Both the single and two target cell models predict that in contrast with seasonal infections which respond best to antiviral treatment when it is administered within 2 dpi, delayed antiviral treatment of infections with more severe influenza strains can be effective in reducing the viral load and shortening the duration of the infection even when administered late.

## Discussion

It has been suggested that differences in disease severity between human- and avian-derived influenza virus infections may in part be due to differences in target cell tropism between the two viral subtypes [16], [22]. In order to determine how cell tropism affects the dynamics of an influenza virus infection, we developed a mathematical model consisting of two different target cell populations: a default and a secondary population. The secondary cell population differs from the default population only in its susceptibility to infection and rate of virus production. The two target cell model is a simple extension of the single target cell model with delayed viral production introduced by Baccam et al. [28].

We found that within a certain area of the parameter space, the viral load in the two target cell infection model quickly rises to high levels and high viral production is maintained over extended periods of time. The two target cell model displays this behaviour when the secondary population is such that 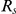 is slightly greater than 1. The infection proceeds when the default cell population falls prey to the infection and is consumed rapidly (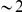). This in turn increases the viral load to levels sufficient to initiate infection of the more refractory secondary cell population. Once infection is established within the secondary population, it is consumed slowly by the virus resulting in a long and severe infection.

In order to determine whether these sustained viral titers are representative of the dynamics observed for severe influenza infections in vivo, and to see whether those dynamics can be captured with reasonable parameter values, we compared our two target cell model's performance to that of the single target cell model by fitting both models to measurements of influenza infections in mice [52] and humans [7]. Fits to data from infections in mice with one of four different strains (two human strains and two avian strains) indicated that the two target cell model was better able to capture the dynamics of the four infections compared to the single target cell model. And while fits of the single target cell model to the experimental data resulted in reasonable fits, these fits were made possible through unrealistic parameter values. When parameters were fixed to more biologically reasonable values, the two target cell model remained in good agreement with the data, but the single target cell model performed poorly. This indicates that the single target cell model is not adequate to capture the dynamics of these infections, and that the two target cell model is more appropriate. However, other extensions of the single target cell model with additional parameter values could likely do as well as the two target cell model. Fits to data of human infections with either human- or avian-derived influenza strains indicated that both the single and two target cell models could fit the data equally well with reasonable parameter values, and the single target cell model was best supported (smaller 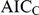) by the limited amount of data available. Overall, the fits suggested that while the two target cell model is more appropriate than the single target cell model to capture a wider range of infection dynamics within reasonable parameter ranges — which is not surprising given its two additional parameters — experimental data was insufficient to reject or confirm the cell tropism hypothesis offered by the two target cell model.

We also used the models parametrized through the fits of the human infection data to explore how the single and two target cell models differed in their predictions of the effectiveness of antiviral treatment of influenza infections with human or avian strains. The predictions from both the single and two target cell models were similar and indicated that while treatment of seasonal influenza infections (human-derived strains) with antivirals is of limited effectiveness when treatment is administered more than 2 dpi, treatment of more severe influenza infections (e.g. avian-derived strains) can be effective even when administered late.

In order to give rise to high viral titers, sustained over several days, the cell tropism hypothesis suggests that, for the parameter values chosen in this paper, the secondary cell population is more difficult to infect than the default one. It further suggests that once infected, these secondary cells produce more virus than cells of the default type. While it is readily acceptable that default and secondary cells have differing infection rates on the basis that differences in cell receptor expressions on different cell populations undoubtedly affect cell-virus binding rates making some cells harder to infect for certain virus strains, the latter requirement is less obvious. It is helpful to recall that through the re-scaling of the parameters (2), it possible to achieve the same kinetics with secondary cells producing as much or even less virus than cells of the default type. In the absence of further information regarding possible differences in viral production rates between the two cell types, it is still interesting to consider what factors could be responsible for such differences. It could be that the genotype which changes the virus' ability to infect a cell population which produces more virus also leaves the virus with an impairment in its ability to be transmitted to another host, say specifically because of its receptor preference. For example, the SA
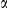
2,3 Gal receptors are predominantly found on the surface of ciliated cells but also in mucin. So while a virus which adapts to target SA
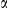
2,3 Gal receptors could have access to supposedly higher viral producing cells (if such cells exist), this would come at the price that many of these virions would be much less readily transmitted to another host due to an increase in their rate of mucus binding at the expense of target cell binding. It is also likely that viral infectivity is not independent of the rate of production of free virions. In the two target cell model the virus production rate, 
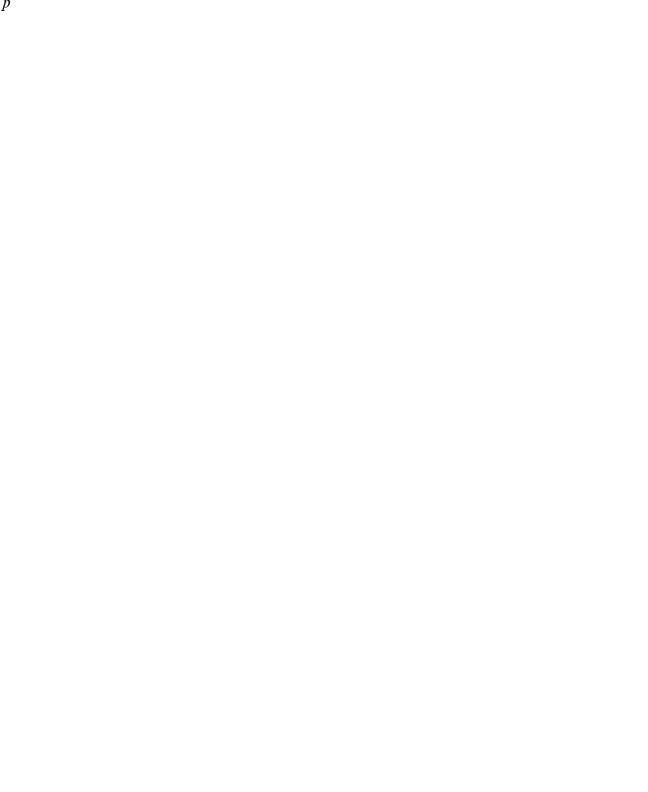
, stands for both virus production and release. If many virions are produced but are not readily released, 
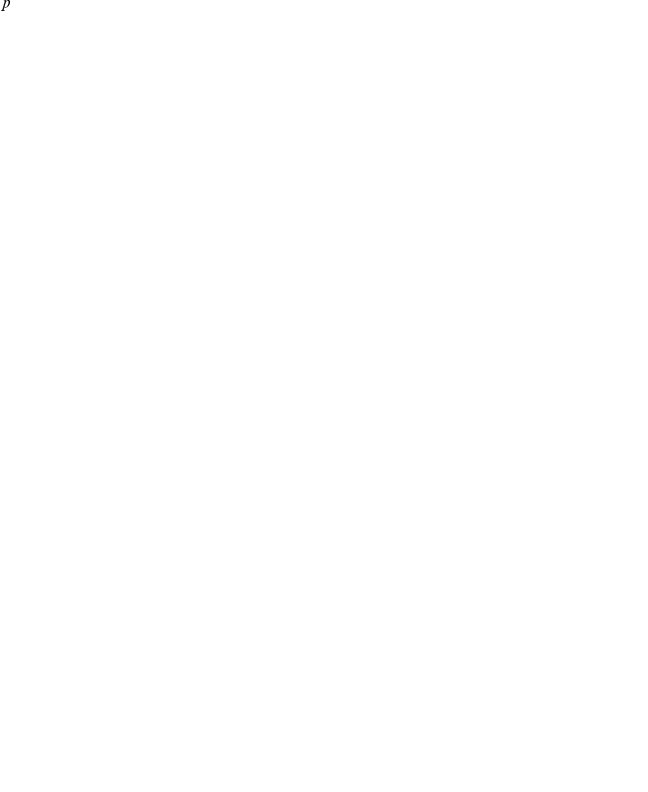
 will effectively be smaller. If a cell is harder to infect, this is likely due to weaker cell-virus receptor binding (weak HA activity) making the virus “less sticky” which would likely results in higher viral release rates even for equivalent viral production rates, and hence an increase in 
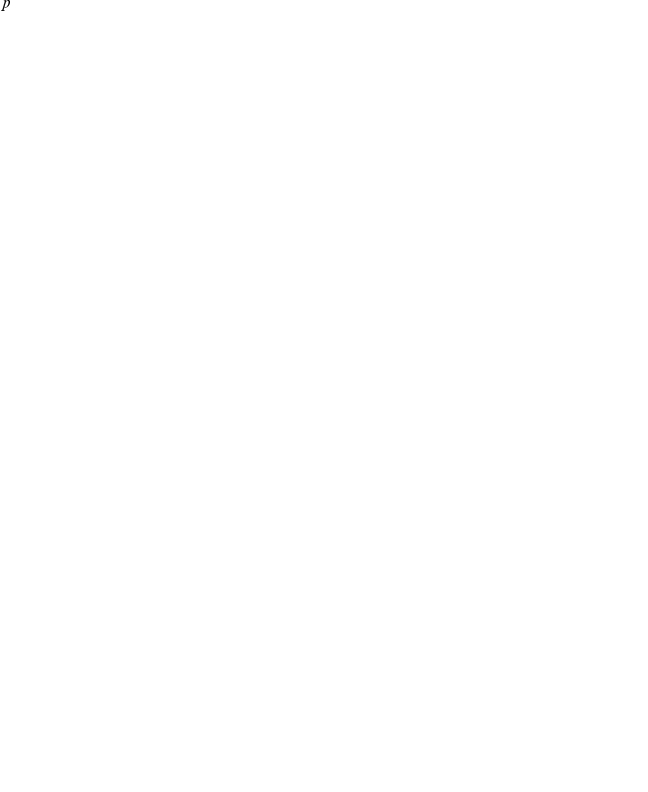
.

While it has been established that cell tropism plays a role in shaping influenza dynamics [53], [54], there is insufficient data at this point to establish whether this role is significant or not when contrasted against the role of other likely factors such as reduced immunity or a virus-induced upregulation of cytokines, in shaping the severity of the infection. If more experimental data, both in terms of quantity (e.g., more viral titer over a longer time period) and diversity (e.g., levels of interferon, antibodies, cell viability) were to become available, one could construct different models incorporating the most likely factors and it may be possible to eliminate some of these factors. In order to evaluate the validity of the predictions made by the two target cell model, it would be useful to have more viral titer measurements during the early phase of the infection. Our model produces viral titer with a kink or bend when the infection in the default cell population reaches a peak which should be visible in experimental data collected sufficiently frequently during this early time. In addition, single-cycle viral yield experiments in the presence of differing ratios of the two cell types would indicate whether, and by how much, viral production rates differ between different cell types for different strains [55].

Regardless of whether or not cell tropism plays a significant role in shaping infection dynamics, we believe that the two target cell model is valuable because it can capture dynamics that the simpler single target cell model cannot with the addition of only two more parameters: the rate of infection of the secondary cells by the virus, and the rate of virus production in these cells. In addition, the nature of these two extra parameters allows them to act as generic stand-ins for a multitude of other effects including a reduced immune response. For example, the secondary cell population could correspond to cells which would not normally get infected (e.g., slightly deeper in the lung) because the immune response would clear the infection before it gets to these cells. In this context, a seasonal influenza infection could be characterized as having an infection rate for the secondary cells which would be negligible compared to that for a more severe influenza infection where infection of these secondary cells would become possible and would be reflected by a larger secondary cells' infection rate, albeit one that is much smaller than that of the default target cell population. The increased viral production rate could simply result from the fact that later in the infection, the immune response begins to contract (e.g., interferon seems to disappear even in the continued presence of infection, see [42]) and as such production of virus late in the infection, i.e., when the secondary cells get infected, would be higher at that time (or in these cells, since at that time only these cells are left) than at earlier times when the immune response was still strong. Thus, we see the two target cell model as a versatile stand-in for a range of phenomena which, in our opinion, can be captured in a generic way by these two parameters.

The two target cell model does not explicitly include an immune response or cell regeneration. This may appear to be a critical oversight given that some of the infections captured by the two target cell model can last up to 16 dpi. Since our analysis focuses solely on viral titers, and not cell numbers, over time, proper consideration of cell regeneration is relevant only if these regenerated cells can participate in the infection and are infectible to the same extent as the original target cell population. This is not necessarily the case. For example in rodents, the epithelium is reformed within 36–48 h, is differentiated on the fourth day, with ciliated cells appearing in 10–14 days, and complete regeneration in 6 weeks [43]. These regeneration times would likely be longer in human lungs such that cell regeneration may not have a significant effect on infection dynamics even when considering long-lasting infections. Another mathematical model which includes cell regeneration to capture influenza infections in mice over the course of 10 days found regeneration times upwards of 67 d suggesting that either cell regeneration did not play a significant role in these infections, or that the effect of cell regeneration could be accounted for implicitly through the other parameters of the model [42]. While we did not present our results here, we did study the effect of incorporating cell regeneration into our two target cell model at a rate of 0.25 d^−1^ [42] and found that it did not significantly affect the dynamics of the model over the course of the main infection (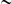 the first 16 days). However, as is often the case when models incorporate cell regeneration, the dynamics on longer time scales was characterized by large, unrealistic, oscillations. This, in our opinion, supports the hypothesis that newly regenerated cells do not participate in the infection in a significant manner or at least have significantly reduced infectibility. The immune response, however, is certainly not negligible and likely plays an important role, especially in later times. Unfortunately, while several publications have attempted to extend the mathematical model for influenza in humans to include an immune response [56]–[58], all were theoretical explorations with insufficient experimental data to support the extensions and parametrize the response. Models incorporating a simple immune response have been used to fit experimental data from animals [42], [59], [60] and can, under some parameter regimes, produce long-lasting viral titers. Thus we feel this is an area to be explored. Unfortunately, in the absence of sufficient experimental data characterizing the immune response in humans, it is not possible to incorporate it into our model in a convincing way. Hopefully, as discussed above, some of the components of the immune response have been incorporated into our model's parameter values in a implicit way.

Finally, given that the two target cell model proposed here explicitly takes into consideration cell tropism, its biggest contribution may be its application to translating results obtained in one host or cell culture system (e.g., mouse, MDCK) into predictions for the course and outcome of infections in another host or cell culture system (e.g., human, ferret, differentiated human epithelium cells). As such, we feel that it will be a very useful tool as more quantitative data about cell infection rates and viral production rates of cells for infections with different influenza strains become available. In addition, since our model is a simple extension of the classic viral dynamics model used to capture in-host infection with a variety of other viruses such as HIV [35], [61], and Hepatitis viruses [37], [38], [62], [63], our conclusions also apply to these other diseases where different cell types can be affected by the virus.

## Supporting Information

Appendix S1 Exploring cell tropism as a possible contributor to influenza infection severity.
(0.45 MB PDF)

## References

[1] Writing Committee of the Second World Health Organization Consultation on Clinical Aspects of Human Infection with Avian Influenza A (H5N1) Virus (2008). Update on avian influenza A (H5N1) virus infection in humans.. N Engl J Med.

[2] Novel Swine-Origin Influenza A (H1N1) Virus Investigation Team (2009). Emergence of a novel swine-origin influenza A (H1N1) virus in humans.. N Engl J Med.

[3] Gallaher WR (2009). Towards a sane and rational approach to management of influenza H1N1 2009.. Virol J.

[4] Kaufman MA, Duke GJ, McGain F, French C, Aboltins C (2009). Life-threatening respiratory failure from H1N1 influenza 09 (human swine influenza).. Med J Aust.

[5] Eizenberg P (2009). The general practice experience of the swine flu epidemic in Victoria — lessons from the front line.. Med J Aust.

[6] Coombes R (2009). Doctors call for guidance on how to prioritise critically ill patients in swine flu pandemic.. BMJ.

[7] de Jong MD, Simmons CP, Thanh TT, Hien VM, Smith GJD (2006). Fatal outcome of human influenza A (H5N1) is associated with high viral load and hypercytokinemia.. Nat Med.

[8] Kaiser L, Fritz RS, Strauss SE, Gubareva L, Hayden F (2001). Symptom pathogenesis during acute influenza: Interleukin-6 and other cytokine responses.. J Med Virol.

[9] Seo SH, Webster RG (2002). Tumor necrosis factor alpha exerts powerful anti-influenza virus effects in lung epithelial cells.. J Virol.

[10] Cheung C, Poon LLM, Lau AS, Luk W, Lau YL (2002). Induction of proinflammatory cytokines in human macrophages by influenza A (H5N1) viruses: A mechanism for the unusual severity of human disease?. Lancet.

[11] Chan M, Cheung C, Chui W, Tsao S, Nicholls J (2005). Proinflammatory cytokine responses induced by influenza A (H5N1) viruses in primary human alveolar and bronchial epithelial cells.. Respir Res.

[12] Hsieh SM, Chang SC (2006). Insufficient perforin expression in CD8+ T cells in response to hemagglutinin from avian influenza (H5N1) virus.. J Immunol.

[13] Seo SH, Hoffmann E, Webster RG (2002). Lethal H5N1 influenza viruses escape host anti-viral cytokine responses.. Nature Med.

[14] Pekosz A, Newby C, Bose PS, Lutz A (2009). Sialic acid recognition is a key determinant of influenza A virus tropism in murine trachea epithelial cell cultures.. Virology.

[15] Stevens J, Blixt O, Chen LM, Donis RO, Paulson JC (2008). Recent avian H5N1 viruses exhibit increased propensity for acquiring human receptor specificity.. J Mol Biol.

[16] Matrosovich MN, Matrosovich TY, Gray T, Roberts NA, Klenk HD (2004). Human and avian influenza viruses target different cell types in cultures of human airway epithelium.. Proc Natl Acad Sci USA.

[17] Matrosovich MN, Matrosovich TY, Uhlendorff J, Garten W, Klenk HD (2007). Avian-virus-like receptor specificity of the hemagglutinin impedes influenza virus replication in cultures of human airway epithelium.. Virology.

[18] Nicholls J, Chan M, Chan W, Wong H, Cheung C (2007). Tropism of avian influenza A H5N1 in the upper and lower respiratory tract.. Nature Medicine.

[19] Shinya K, Ebina M, Yamada S, Ono M, Kasai N (2006). Influenza virus receptors in the human airway.. Nature.

[20] Thompson CI, Barclay WS, Zambon MC, Pickles RJ (2006). Infection of human airway epithelium by human and avian strains of influenza A virus.. J Virol.

[21] van Riel D, Munster VJ, de Wit E, Rimmelzwaan GF, Fouchier RA (2006). H5N1 virus attachment to lower respiratory tract.. Science.

[22] Ibrecevic A, Pekosz A, Walter MJ, Newby C, Battaile JT (2006). Influenza virus receptor specificity and cell tropism in mouse and human airway epethilial cells.. J Virol.

[23] Kogure T, Suzuki T, Takahashi T, Miyamoto D, Hidari KI (2006). Human trachea primary epithelial cells express both sialyl(*α*2–3)Gal receptor for human parainfluenza virus type 1 and avian influenza viruses, and sialyl(*α*2–6)Gal receptor for human influenza virus.. Glycoconj J.

[24] Oh DY, Barr IG, Mosse JA, Laurie KL (2008). MDCK-SIAT1 cells show improved isolation rates for recent human influenza viruses compared to conventional MDCK cells.. J Clin Microbiol.

[25] Ning ZY, Luo MY, Qi WB, Yu B, Jiao PR (2009). Detection of expression of influenza virus receptors in tissues of BALB/c mice by histochemistry.. Vet Res Commun.

[26] Glaser L, Conenello G, Paulson J, Palese P (2007). Effective replication of human influenza viruses in mice lacking a major *α*2,6 sialyltransferase.. Virus Res.

[27] van Riel D, Munster VJ, de Wit E, Rimmelzwaan GF, Fouchier RA (2007). Human and avian influenza viruses target different cells in the lower respiratory tract of humans and other mammals.. Am J Pathol.

[28] Baccam P, Beauchemin C, Macken CA, Hayden FG, Perelson AS (2006). Kinetics of influenza A virus infection in humans.. J Virol.

[29] Beauchemin C, McSharry J, Drusano G, Nguyen J, Went G (2008). Modeling amantadine treatment of influenza A virus in vitro.. J Theor Biol.

[30] Handel A, Longini IM, Antia R (2007). Neuraminidase inhibitor resistance in influenza: Assessing the danger of its generation and spread.. PLoS Comp Biol.

[31] Diekmann O, Heesterbeek JAP, Metz JAJ (1990). On the definition and the computation of the basic reproduction ratio *R*
_0_ in models for infectious diseases in heterogeneous populations.. J Math Biol.

[32] Ganusov VV, Bergstrom CT, Antia R (2002). Within-host population dynamics and the evolution of microparasites in a heterogeneous host population.. Evolution.

[33] Hethcote HW, van Ark JW (1987). Epidemiological models for heterogeneous populations: Proportionate mixing, parameter estimation and immunization programs.. Math Biosci.

[34] Larson RC (2007). Simple models of influenza progression within a heterogeneous population.. Op Res.

[35] Perelson AS, Essunger P, Cao Y, Vesanen M, Hurley A (1997). Decay characteristics of HIV-1 infected compartments during combination therapy.. Nature.

[36] Bajaria SH, Webb G, Cloyd M, Kirschner D (2002). Dynamics of naive and memory CD4+ T lymphocytes in HIV-1 disease progression.. JAIDS.

[37] Payne RJH, Nowak MA, Blumberg BS (1992). Analysis of a cellular model to account for the natural history of infection by the hepatitis B virus and its role in the development of primary hepatocellular carcinoma.. J Theor Biol.

[38] Payne RJH, Nowak MA, Blumberg BS (1994). A cellular model to explain the pathogenesis infection by the hepatitis B virus.. Math Biosci.

[39] Dahari H, Feliu A, Garcia-Retortillo M, Forns X, Neumann AU (2005). Second hepatitis C replication compartment indicated by viral dynamics during liver transplantation.. J Hepatol.

[40] Eaton JW (2008). GNU Octave version 3.0.1. A free open-source software for solving linear and nonlinear problems numerically, and for performing other numerical experiments using a language that is mostly compatible with Matlab.. http://www.octave.org.

[41] Hindmarsh A, Stepleman R (1983). ODEPACK, a systematized collection of ODE solvers.. IMACS Transactions on Scientific Computation.

[42] Handel A, Longini IM, Antia R (2010). Towards a quantitative understanding of the within-host dynamics of influenza A infections.. J R Soc Interface.

[43] Crystal RG, West JB (1991). The Lung: Scientific Foundations.

[44] Seber GAF, Wild CJ (1989). Nonlinear Regression.

[45] Matrosovich MN, Matrosovich TY, Gray T, Roberts NA, Klenk HD (2004). Neuraminidase is important for the initiation of influenza virus infection in human airway epithelium.. J Virol.

[46] Yamada S, Suzuki Y, Suzuki T, Le MQ, Nidom CA (2006). Hemagglutinin mutation responsible for the binding of H5N1 influenza A viruses to human-type receptors.. Nature.

[47] van den Driessche P, Watmough J (2008). Further notes on the basic reproductive number.. Mathematical Epidemiology.

[48] Nowak MA, May RM (2000). Virus Dynamics: Mathematical Principles of Immunology and Virology.

[49] Hayden FG, Fritz RS, Lobo MC, Alvord WG, Strober W (1998). Local and systemic cytokine responses during experimental human influenza A virus infection.. J Clin Invest.

[50] Fritz RS, Hayden FG, Calfee DP, Cass LMR, Peng AW (1999). Nasal cytokine and chemokine response in experimental influenza A virus infection: Results of a placebo-controlled trial of intravenous zanamivir treatment.. J Infect Dis.

[51] de Jong MD, Cam BV, Qui PT, Hien VM, Thanh TT (2005). Fatal avian influenza A (H5N1) in a child presenting with diarrhea followed by coma.. N Engl J Med.

[52] Perrone LA, Plowden JK, García-Sastre A, Katz JM, Tumpey TM (2008). H5N1 and 1918 pandemic influenza virus infection results in early and excessive infiltration of macrophages and neutrophils in the lungs of mice.. PLoS Pathog.

[53] Nicholls J, Chan R, Russell R, Air G, Peiris J (2008). Evolving complexities of influenza virus and its receptors.. Trends in Microbiology.

[54] Li I, Chan K, To K, Wong S, Ho P (2009). Differential susceptibility of different cell lines to swine-origin influenza A H1N1, seasonal human influenza A H1N1, and avian influenza A H5N1 viruses.. J Clin Virol.

[55] Holder BP, Beauchemin CAA (2010). Exploring the effect of implementing different biological delays in constructing kinetic models of influenza infection within a host or cell culture.. BMC Public Health.

[56] Bocharov GA, Romanyukha AA (1994). Mathematical model of antiviral immune response III. Influenza A virus infection.. J Theor Biol.

[57] Lee HY, Topham DJ, Park SY, Hollenbaugh J, Treanor J (2009). Simulation and prediction of the adaptive immune response to influenza A virus infection.. J Virol.

[58] Hancioglua B, Swigona D, Clermont G (2007). A dynamical model of human immune response to influenza A virus infection.. J Theor Biol.

[59] Miao H, Hollenbaugh JA, Zand MS, Holden-Wiltse J, Mosmann TR (2010). Quantifying the early immune response and adaptive immune response kinetics in mice infected with influenza A virus.. J Virol.

[60] Saenz RA, Quinlivan M, Elton D, MacRae S, Blunden AS (2010). Dynamics of influenza virus infection and pathology.. J Virol.

[61] Perelson AS, Neumann A, Markowitz M, Leonard J, Ho D (1996). HIV-1 dynamics *in vivo*: Virion clearance rate, infected cell life-span, and viral generation time.. Science.

[62] Neumann AU, Lam NP, Dahari H, Gretch DR, Wiley TE (1998). Hepatitis C viral dynamics in vivo and the antiviral efficacy of interferon-*α* therapy.. Science.

[63] Nowak M, Bonhoeffer S, Hill A, Boehme R, Thomas H (1996). Viral dynamics in hepatitis B viral infection.. Proc Natl Acad Sci USA.

