# Supplementary material for: Exploring Cell Tropism as a Possible Contributor to Influenza Infection Severity
Source: PLoS One. 2010 Nov 23;5(11):e13811. doi: 10.1371/journal.pone.0013811 (PMC2990709; doi:10.1371/journal.pone.0013811)
Supplement: Appendix S1 — Exploring cell tropism as a possible contributor to influenza infection severity. (0.45 MB PDF) [file pone.0013811.s001.pdf]

Appendix S1:

Exploring cell tropism as a possible contributor to influenza infection severity

Hana M. Dobrovolny[1,†], Marc J. Baron[1,†], Ronald Gieschke[2], Brian E. Davies[3], Nelson L. Jumbe[2], and Catherine A.A. Beauchemin[1,*]

[1] **Department of Physics, Ryerson University, Toronto, Ontario, Canada**
[2] **F. Hoffmann-La Roche Ltd., Basel, Switzerland**
[3] **F. Hoffmann-La Roche Inc., Nutley, New Jersey, United States of America**
[†] **These authors contributed equally to this manuscript.**
[*] **Corresponding author. Address: Department of Physics, Ryerson University, 350 Victoria St., Toronto, ON, M5B 2K3, Canada. E-mail: cbeau@ryerson.ca**

A Additional measures of disease severity

To assess the effect of different parameters on the severity of an infection, we introduced some measures of disease severity in the main body of the paper, but there are other measures that can characterize the severity of the infection. In this appendix, we discuss measures of disease severity that can be measured or derived from the model itself, but may be difficult to measure or estimate clinically from measurements of the viral titer alone.

A.1 Fraction of dead cells

The amount of destruction to the epithelium of the respiratory tract may determine whether patients suffer complications and is also an indicator of how long it may take for the patient to recover from the disease. Our chosen parameters lead to the death of all default cells, however the number of secondary cells that die in the course of the infection will vary as r_p and r_β vary (Figure 1(a)). Few secondary cells participate in the infection when r_p and r_β are small (or $R_s < 1$), so there is little death of the secondary cells. While R_s is below 1, the fraction of dead secondary cells shows a strong dependence on r_β — as r_β decreases, the fraction of dead secondary cells increases since they are now easier to infect. In this region, the infection cannot be sustained in the secondary cells due to their low production rate, but there can still be substantial death of the secondary population from the virus produced by infection of the default population. The fraction of dead cells shows a very abrupt change at $R_s = 1$, with both cell populations dying when $R_s > 1$.

A.2 Basic reproductive number

Since our model consists of a heterogeneous cell population, R_s and R_d are not, strictly speaking, the basic reproductive numbers of the two cell populations. However, they do provide a measure of whether an infection is sustainable within either population and how widely it might spread within that population. Again, the behaviour of the default population is independent of r_p and r_β, so R_d is not dependent on r_p and r_β. R_s depends linearly on both r_p and r_β and so increases as we move from the bottom left corner to the upper right corner (Figure 1(b)).

A.3 Largest eigenvalue

The largest eigenvalue at the start of the infection is a measure of how quickly the infection grows. Below and slightly above $R_s = 1$ (black line in Figure 1(c)), the initial growth rate of the infection is determined solely by the growth of the infection in the default cell population which is independent of r_p and r_β. Recall that even when R_s is slightly greater than one, the secondary cell population does not participate in the infection until there is enough virus produced to trigger the infection in the secondary population,

so it does not contribute to the initial growth of the infection. As r_p and r_β increase, the secondary cell population becomes involved earlier and we see the initial growth rate increase as these cells become infected and start to produce virus at the start of the infection.

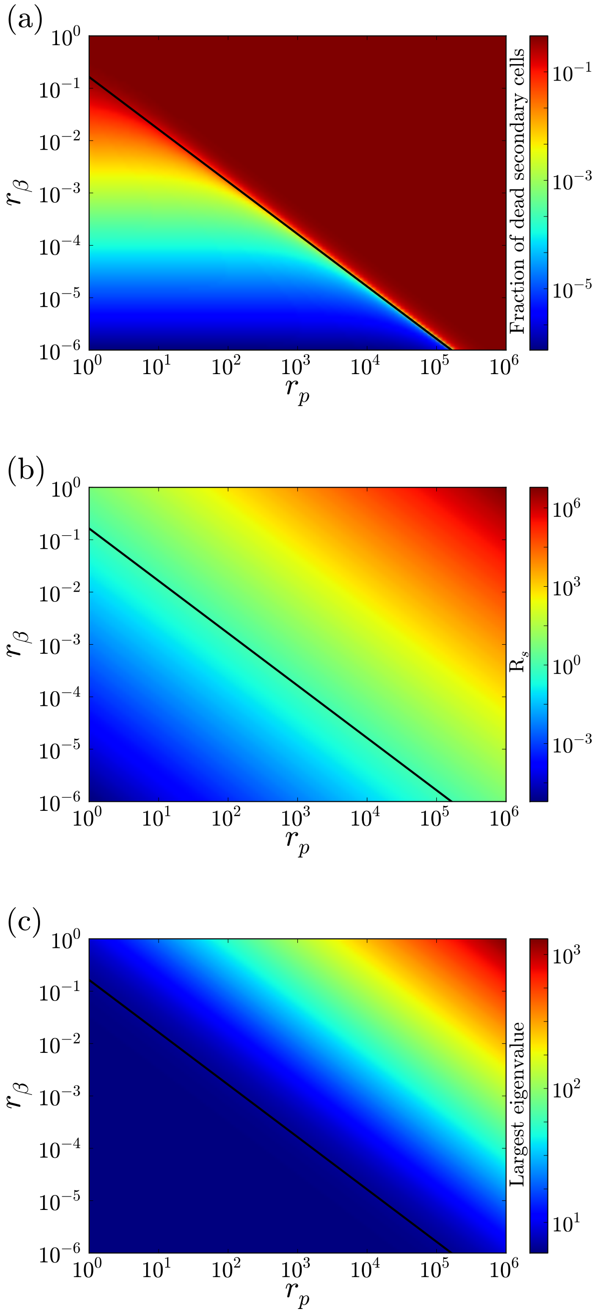

Figure 1. Additional measures of disease severity for varying properties of the secondary cell population. The effect of varying the secondary cell's susceptibility to infection (r_β) and their rate of virus production (r_p) compared to that for cells of the default type on different measures of disease severity: (a) the fraction of dead secondary cells, (b) the basic reproductive number of the secondary population (R_s) and (c) the largest eigenvalue at the start of the infection. Note that the value of the disease severity measures in the top left corner of each graph ($r_\beta, r_p = 1,1$) corresponds to the single target cell model. All other parameters are set as specified in Table 1 of the paper, with $r_T = 0.5$. The stability condition $R_s = 1$ is also indicated (black line).
